# Floristic Diversity, Structure and Carbon Storage of a Sub-Andean Forest in Southwestern Colombia: The Case of the El Mangón

**DOI:** 10.3390/biology15141154

**Published:** 2026-07-15

**Authors:** Luis Eduardo López-Vargas, Diego Jesús Macías-Pinto, Jhoy Fleming Córdoba-Calvo, Jorge Hernan Patiño Rodriguez

**Affiliations:** 1 SACHAWAIRA Plant Diversity Research Group, GELA—Latin American Ethnobotanical Group, Department of Biology, Faculty of Natural, Exact Sciences and Education, Universidad del Cauca, Popayán 190003, Cauca, Colombia; djmacias@unicauca.edu.co (D.J.M.-P.); jhoycordoba@unicauca.edu.co (J.F.C.-C.); 2 Bogotá D.C. Biotechnology and Environment Research Group, Department of Biology, Faculty of Engineering, Administration and Basic Sciences, Universidad INCCA de Colombia, Bogotá 110110, Colombia; jhpatinor@unincca.edu.co

**Keywords:** sub-Andean forest, floristics, aboveground carbon, plant diversity, hill numbers, Colombia, conservation, Colombian Andes

## Abstract

Sub-Andean forests of the Colombian Central Cordillera are among the most biodiverse and threatened Neotropical ecosystems, yet their floristic composition, vegetation structure, and aboveground carbon storage remain poorly documented. We characterized the floristic diversity, vegetation structure, and aboveground carbon storage of the El Mangón sub-Andean forest remnant (24 ha; 1600–1700 m a.s.l., Cauca, Colombia) using general (non-systematic) collections across the total area and a structural inventory in five 50 × 4 m transects (498 individuals, 35 species). A total of 281 species, 209 genera, and 99 families were recorded; epiphytes represented 44.13% of species, exceeding typical values (25–35%) for this forest type. Diversity indices were intermediate (H′ = 2.55; DMg = 5.47; 1-D = 0.89). *Palicourea crocea* dominated structurally (IVI = 45.12), whereas aboveground carbon—re-estimated with the pantropical model (total = 72.85 Mg C ha^−1^)—was concentrated in a few large canopy trees (*Alchornea latifolia*, 32%; *Myrsine guianensis*, 16%), with *P. crocea* contributing only 3.6%. Three novel carbon indices (CVI, CCEI, CSI) integrate storage magnitude with capture efficiency and spatial stability; the CSI differed significantly among spatial-distribution groups (Kruskal–Wallis, *p* = 0.003), being higher in aggregated, large-stemmed species than in low-aggregation species. El Mangón ranks among the most diverse sub-Andean remnants documented to date in southwestern Colombia, underscoring its conservation priority in an increasingly fragmented landscape.

## Simple Summary

Tropical mountain forests in the Colombian Andes are among the most species-rich places on Earth, yet many small forest patches that survive inside farmland have never been studied. We examined one such patch, called El Mangón, in southwestern Colombia: a forest of about 24 hectares that one family has protected since 1912. During eight months of fieldwork, we listed all the plants we could find and, in smaller measured plots, recorded the size and position of trees and shrubs to estimate how much carbon the vegetation stores above the ground. We found an unusually rich flora of 281 kinds of plants, including a remarkable number that grow perched on tree branches, together with mosses and lichens. The forest is still young and recovering, with many small stems and only a few tall trees, so the amount of carbon stored in its wood is modest. We also propose simple ways to rank species by how much carbon they hold and by how evenly that carbon is spread across the forest. Our results show that even small, privately protected forests can shelter exceptional plant diversity, and they support the local effort to declare El Mangón a protected nature reserve.

## 1. Introduction

Sub-Andean forests constitute one of the most biodiverse and threatened ecosystems in the Neotropics. Distributed between 1000 and 2400 m a.s.l. in Colombia, they harbor exceptional levels of species richness and endemism, function as biological corridors among altitudinal zones, and provide critical ecosystem services, including hydrological regulation, soil retention, and carbon storage [1,2]. In Colombia, these forests have lost more than 74% of their original cover because of agricultural-frontier expansion, selective timber extraction, and land-use conversion [3]. The most recent national monitoring data indicate that 113,608 ha were deforested in Colombia during 2024 [4], and that in the department of Cauca, forest loss reached 790.34 ha that same year [5], a trend that severely affects connectivity among fragments and alters species composition in sub-Andean landscapes [6]. The Colombian Central Cordillera accounts for an especially significant fraction of this threatened diversity: its sub-Andean forests are dominated by families such as Lauraceae, Melastomataceae, Rubiaceae, and Piperaceae [7,8,9], and represent one of the systems with the greatest floristic-documentation deficit in the country [2,10].

Despite recognition of their ecological importance, knowledge of the floristic diversity and structure of the sub-Andean forests of the Colombian Central Cordillera remains fragmentary. Available studies in the region document between 62 and 431 species per site, with methodological differences that hinder direct comparison [11,12,13,14,15], and only a few simultaneously integrate floristic characterization, quantitative structural analysis, and carbon-storage estimation [16]. This gap is particularly relevant given that montane secondary forests accumulate between 40 and 70% of the carbon stored in comparable mature forests [17,18] and play an increasing role in national climate-change mitigation strategies. The development of functional indices that integrate carbon storage with individual capture efficiency and the stability associated with species spatial-distribution patterns represents a scarcely explored approach in Colombian floristic studies, with direct potential to guide conservation and restoration strategies based on functional efficiency.

The El Mangón forest remnant, located in the corregimiento of Tunía, municipality of Piendamó, department of Cauca (1600–1700 m a.s.l.; 24 ha), constitutes a case of special interest for the landscape ecology of fragmented areas of the Central Cordillera. With an uninterrupted history of private conservation since 1912, when the Gómez family decided to protect the forest in a context of growing agricultural pressure, this remnant represents a rare example of a sub-Andean forest with low direct intervention embedded in an intensive agricultural mosaic. The ongoing initiative to declare the property a Natural Reserve of Civil Society (Reserva Natural de la Sociedad Civil, RNSC) confers immediate relevance on its floristic and functional characterization for private-conservation policy in Colombia. Until now, no scientific study had comprehensively documented its floristic diversity, vegetation structure, or aboveground carbon-storage capacity.

The general objective of this study was to characterize the floristic diversity, vegetation structure, and carbon storage of the El Mangón sub-Andean forest, in order to contribute to the knowledge of the sub-Andean ecosystems of the Colombian Central Cordillera and to provide baseline information for their conservation and management. Specific objectives were: (i) to inventory and analyze the taxonomic composition of the remnant through general (non-systematic) collections across the total area (24 ha); (ii) to characterize the horizontal and vertical structure of the forest through systematic transect-based sampling; (iii) to estimate aboveground biomass and carbon storage by species and to interpret the results using the Carbon Valuation Index (CVI), the Carbon Capture Efficiency Index (CCEI) and the Carbon Sustainability Index (CSI), which integrate storage magnitude with individual efficiency and the spatial stability of species; and (iv) to compare the findings with analogous studies in sub-Andean forests of southwestern Colombia and the Central Cordillera.

## 2. Materials and Methods

### 2.1. Study Area

**Figure 1:**
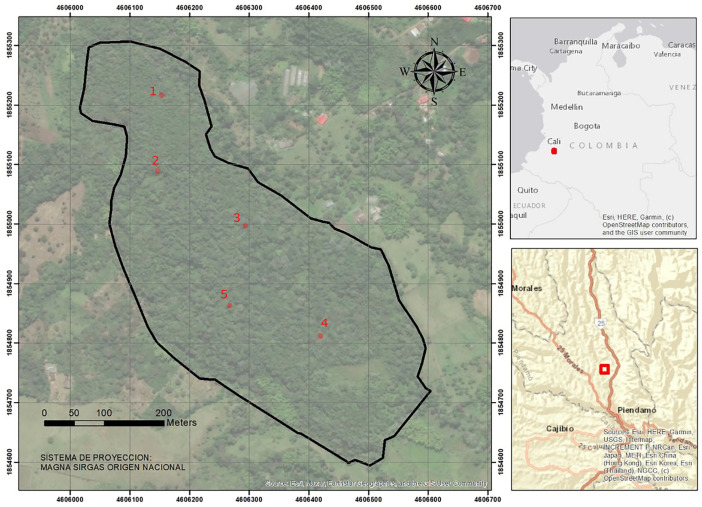
Geographic location of the El Mangón sub-Andean forest remnant in the corregimiento of Tunía, municipality of Piendamó, department of Cauca, southwestern Colombia (1600–1700 m a.s.l., 24 ha). The red square indicates the sampling area located in the municipality of Piendamó, corregimiento of Tunía. The Inset shows the position of the department of Cauca within Colombia. The five 50 × 4 m structural transects (Component II) are marked and numbered (1–5) within the remnant. Source: Own elaboration using QGIS software (version 3.34), based on Esri basemaps and satellite imagery, with data from Esri, Maxar, Earthstar Geographics, HERE, Garmin, USGS, Intermap, INCREMENT P, NRCan, OpenStreetMap contributors, and the GIS User Community.

The El Mangón forest remnant is in the corregimiento of Tunía, municipality of Piendamó, department of Cauca, Colombia (2º40′52.2″ N, 76º32′25.5″ W), covering an area of 24 ha between 1600 and 1700 m a.s.l. The site has a mean annual temperature of 18 °C and an annual precipitation of 2300 mm. It is crossed by three water bodies (Colcha, Espino, and Quebrada Grande streams) and contains a water spring in its interior (Figure 1). The conservation history dates to 1912, when the Gómez family decided to protect the forest in the face of growing agricultural pressure in the region, an initiative sustained until the present and oriented toward declaring the property a Natural Reserve of Civil Society (RNSC). 

### 2.2. Study Design and Component Distinction

This study comprises two methodologically differentiated components. Component I (floristic inventory) consisted of documenting total diversity through general (non-systematic) collections across the 24 ha, recording 281 species in 209 genera and 99 families (Appendix A). Component II (structural and functional analysis) was based on systematic sampling in fixed-area transects: 35 species and 498 individuals in 0.1 ha (five 50 × 4 m transects). All alpha diversity, horizontal and vertical structure, biomass, and carbon analyses correspond exclusively to Component II, unless otherwise indicated.

### 2.3. General (Non-Systematic) Floristic Inventory (Component I)

Twelve field expeditions were conducted between June 2025 and January 2026 under botanical collection permit No. 001040 ANLA (granted 30 May 2025), following the general (non-systematic) collection methodology for floristic inventories [19]. Each expedition involved a team of two to four botanical collectors, with an average duration of approximately eight hours each (≈96 expedition-hours; ≈192–384 person-hours for teams of two to four collectors). Collections covered the total area of the remnant (24 ha), including previously inaccessible interior forest zones. Vascular plants, bryophytes, and lichenized fungi (lichens) were recorded, with emphasis on epiphytes, lianas, and ferns; lichens and bryophytes were documented opportunistically as part of the cryptogamic flora. Taxonomic identification was performed using: (a) dichotomous keys and monographs of the flora of Colombia [2,7]; (b) reference collections of the CAUP Herbarium (Universidad del Cauca); (c) virtual collections of GBIF and COL; and (d) nomenclatural verification in World Flora Online (WFO) [20]. Reference specimens were deposited at the CAUP Herbarium under collection numbers Lelopez-4000 to Lelopez-4281.

### 2.4. Structural Sampling via Transects (Component II)

For the structural analysis, five 50 × 4 m transects (total area = 0.1 ha; sampling intensity = 0.42% of the remnant area) were systematically distributed in the interior of the forest, separated from each other by a minimum of 10 m. In each transect, all arboreal and shrubby individuals with circumference at breast height (CBH) ≥ 5 cm (equivalent to DBH ≥ 1.59 cm) were recorded. For each individual we measured: CBH (measuring tape ± 1 mm), total height (Ht) and stem height (Hf) using a Suunto clinometer, and relative spatial coordinates within the transect. Because this structural inventory covers 0.1 ha (0.42% of the remnant), the horizontal structure, biomass and carbon results are interpreted as a first quantitative approximation for the forest interior rather than as a stand-level census; sampling sufficiency for community-level patterns is evaluated by rarefaction/extrapolation and pooled sample coverage (Section Structural Sampling Completeness: Rarefaction and Asymptotic Estimation). Transects were placed in the forest interior (the least-disturbed portion of the remnant), spanning local variation in proximity to the three streams and in canopy-gap conditions; edge and recently disturbed zones were not sampled, so the structural results characterize interior conditions and are expected to under-represent edge-associated and early-pioneer assemblages.

DBH (cm) = CBH (cm)/π(1)

Conversion of circumference to diameter at breast height, applied uniformly to all recorded individuals, is done according to Equation (1).

To evaluate the completeness of the structural inventory, we performed: (a) individual-based rarefaction curves by transect, standardized to the smallest sampling unit size (*n* = 52 individuals; transect 1); and (b) asymptotic diversity estimation using Hill numbers (q = 0, 1 and 2) with 95% confidence intervals computed by bootstrap resampling (999 iterations) using the iNEXT package [21] in R [22].

### 2.5. Alpha Diversity and Horizontal Structure (Component II)

Standard alpha diversity indices were calculated for the 35 species and 498 individuals recorded in the transects, following standard formulations [19]:

*H′* = −∑(*pi* · ln *pi*) (2)

Shannon–Wiener; *pi* = proportion of individuals of species i.

DMg = (*S* − 1)/ln *N* (3)

Margalef; *S* = number of species; *N* = number of individuals.

1 − *D* = 1 − ∑(*pi*^2^) (4)

Simpson (complement); *pi* = proportion of individuals of species i.

*J′* = *H′*/ln *S* (5)

Pielou’s evenness; *S* = number of species.

*d* = *N*max/*N* (6)

Berger–Parker; *N*max = individuals of the most abundant species; *N* = total individuals.

The Importance Value Index (IVI) was calculated as the sum of relative abundance, relative frequency, and relative dominance of each species, with a theoretical maximum of 300 [19]. The spatial-distribution pattern was evaluated using the Pielou aggregation index:

*G*a = *D*o/*D*e (7)

*G*a = aggregation index; *D*o = observed density; *D*e = expected density under random distribution. *G*a < 1: tendency toward dispersion; 1 ≤ *G*a ≤ 2: tendency toward clumping; *G*a > 2: aggregated pattern.

The mixture coefficient was calculated as CM = (number of species/number of individuals) [7].

### 2.6. Vertical Structure and Allometric Analysis (Component II)

Vertical structural diversity was evaluated using the Pretzsch Index (A) [23]:

*A* = −∑(*pij* · ln *pij)*/ln(*S* × *Z*) (8)

where *pij* = proportion of individuals of species i in stratum j; *S* = number of species; *Z* = number of strata.

Vertical strata were defined at four levels—lower stratum (0–8 m), middle stratum (8.1–12 m), upper stratum (12.1–18 m), and emergent stratum (18.1–33 m)—based on the observed total-height distribution of the sampled individuals. Class width for height and diameter distributions was determined using Scott’s criterion [24]:

*h* = 3.5 · *σ* · *n*^−1/3^ (9)

where *h* = class width; *σ* = standard deviation; *n* = number of individuals.

The Ogawa diagram [25] was constructed to visualize height distribution in relation to diameters, and the allometric relationship between Ht and Hf was evaluated using linear regression. Residual normality (Shapiro–Wilk) and homoscedasticity (Breusch–Pagan) assumptions were verified; R^2^ and *p*-values are reported.

### 2.7. Biomass and Carbon Estimation (Component II)

Aboveground biomass (AGB) was estimated using the FAO volumetric methodology [26]:

AGB = VCC × WD × BEF(10)

where AGB = aboveground biomass (t·ha^−1^); VCC = stem volume with bark (m^3^·ha^−1^); WD = wood density (t·m^−3^); BEF = biomass expansion factor.

VCC = BA × *H*f × 0.7 (11)

where BA = basal area (m^2^·ha^−1^); *H*f = mean stem height (m); 0.7 = form factor.

WD = 0.6 t·m^−3^ was used as the regional reference value for tropical Americas [26], and BEF = 1.74. Carbon content was estimated as C = AGB × 0.47 (IPCC convention [27]). Analyses were performed exclusively on Component II and carbon results are expressed in Mg C ha^−1^ (and in kg ha^−1^ at the species level). Because the volumetric route markedly under-estimated stand biomass (implied basal area was inconsistent with the measured 22.6 m^2^ ha^−1^), aboveground biomass was re-estimated with the pantropical allometric model of [28] from individual diameter, total height and wood density; this DBH-based estimate (72.85 Mg C·ha^−1^) is adopted as the primary carbon value.

#### Proposed Carbon Indices (Component II)

To integrate carbon storage with the structural ecological importance of each species, three novel functional indices are proposed in this study: the Carbon Valuation Index (CVI), the Carbon Capture Efficiency Index (CCEI), and the Carbon Sustainability Index (CSI).

CVI*i* = IVI*i* × (*Ci*/*C*total) (12)

where CVI = Carbon Valuation Index; IVI*i* = IVI of species i; *Ci* = carbon stored by species i (kg·ha^−1^); *C*total = total carbon in the sampling area (kg·ha^−1^).

CCEI*i* = *Ci*/(IVI*i* × *Di*) (13)

where CCEI = Carbon Capture Efficiency Index; *Ci* = carbon stored by species i (kg·ha^−1^); IVI*i* = Importance Value Index of species i; *Di* = density of species i (ind·ha^−1^).

For CSI estimation, a spatial factor (*F*spatial) was assigned to each species according to its distribution pattern (Table 1):

CSI*i* = *Ci* × *F*spatial,*i* × (1 + |*H′i*|) (14)

where CSI*i* = Carbon Sustainability Index of species i; *Ci* = stored carbon (kg·ha^−1^); *F*spatial,*i* = spatial factor (Table 1); *H′i* = individual contribution of species i to the community Shannon index (absolute value).

The CSI of group g is calculated as the sum of the individual CSIs of all species in the group: CSIg = ∑CSI*i* for all species in group g.

Each index addresses a management-relevant question that IVI, biomass, carbon or ordination do not answer individually: the CVI identifies species combining high structural importance with a large stored-carbon pool; the CCEI is an intensive (per-importance, per capita) measure that flags efficient storers, subject to a native-status filter; and the CSI weights each species’ carbon by its spatial pattern and diversity contribution as a first-order proxy for how spatially buffered that carbon is. The three indices are descriptive, single-site, single-time tools rather than validated predictors and rest on a single regional wood-density value (Section 2.7), so species-level scores are provisional.

### 2.8. Collection Permits and Ethical Declarations

Biological material collection was carried out under the Framework Collection Permit for Biological Specimens for Non-commercial Scientific Research, Permit 001040 of 30 May 2025, ANLA (Autoridad Nacional de Licencias Ambientales), in accordance with Resolution 1484 of 2014 of the Colombian Ministry of Environment and Sustainable Development (MADS). Specimens were deposited at the CAUP Herbarium of Universidad del Cauca under collection numbers Lelopez-4000 to Lelopez-4281.

### 2.9. Statistical Analysis

Data were organized in Microsoft Excel 2019. Statistical and ecological analyses were performed in R v4.3.0 [22], with packages vegan v2.6–4 [29], ggplot2 v3.4.0 [30], dplyr v1.1.2 [31], and iNEXT R package, version 3.0.1 [21]. Carbon functional groups were defined by k-means partitioning into four groups (Euclidean distance on standardized variables). Principal Component Analysis (PCA) was applied to ten standardized variables (mean = 0, SD = 1): biomass, carbon, IVI, relative abundance, relative frequency, relative dominance, Shannon and Simpson contributions, Pielou aggregation index (Ga), and density. With the corrected (Chave-based) carbon and biomass, PC1 explained 59.4% of the total variance and PC2 explained 20.2% (cumulative variance: 79.6%); the four-group structure and variable–axis associations were preserved when *Palicourea crocea* was excluded (PC1 = 57.4%, PC2 = 32.8%), indicating that both components adequately summarize the structural, ecological, and functional variation among the analyzed species. Comparison of CSI values among spatial-pattern groups was performed using the non-parametric Kruskal–Wallis test, with post hoc pairwise Wilcoxon comparisons and Benjamini–Hochberg correction. Statistical significance was set at α = 0.05.

## 3. Results

Results are presented in two differentiated blocks reflecting the two methodological components of the study (see Section 2.2). Component I corresponds to the general (non-systematic) floristic inventory (24 ha): 281 species, 209 genera, 99 families. Component II corresponds to the structural sampling in five 50 × 4 m transects (0.1 ha): 35 species and 498 individuals. Unless otherwise indicated, all numerical values for IVI, diversity indices, biomass, and carbon refer to Component II.

### 3.1. Taxonomic Diversity and Floristic Richness (Component I—24 ha)

A total of 281 species distributed in 209 genera and 99 families were recorded in the El Mangón forest remnant (Table A1). The species/genus ratio (1.34), species/family ratio (2.84), and genus/family ratio (2.11) indicate a balanced taxonomic structure. The most diverse families were Poaceae (20 spp.), Asteraceae (13 spp.), and Orchidaceae (13 spp.)—16.4% of total richness—which together with Polypodiaceae and Rubiaceae (10 spp. each), Piperaceae (9 spp.), Fabaceae and Melastomataceae (8 spp. each), and Bromeliaceae and Bryaceae (7 spp. each) accounted for 37.4% of species diversity (Table 2). The class distribution showed a predominance of Magnoliopsida (117 spp., 41.64%), followed by Liliopsida (53 spp., 18.86%) and Bryopsida (48 spp., 17.08%). Lecanoromycetes and Polypodiopsida contributed 24 spp. (8.54%) and 25 spp. (8.90%), respectively.

#### Growth Forms—General (Non-Systematic) Floristic Inventory (Component I)

Analysis of growth forms showed that epiphytes constituted the most diverse group with 124 spp. (44.13%), followed by herbs with 97 spp. (34.52%), shrubs with 31 spp. (11.03%), and trees with 25 spp. (8.90%). Hemiparasites and lianas were the least represented, with 2 spp. each (0.71%). The high proportion of epiphytes exceeded the typical values of 25–35% reported for Neotropical sub-Andean forests.

### 3.2. Alpha Diversity Indices (Component II—Transects, 35 spp., 498 ind.)

**Figure 2:**
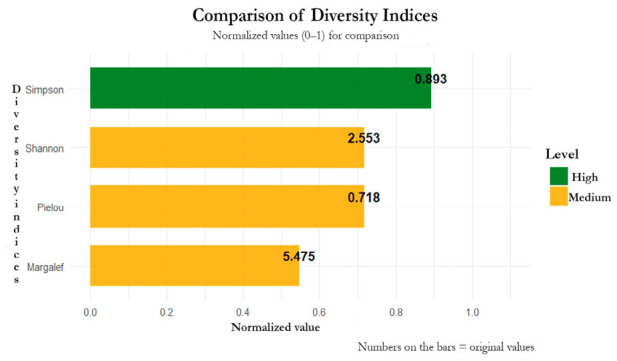
Comparison of alpha diversity indices computed for the El Mangón forest remnant (Component II; *n* = 498 individuals; 35 species). Bars show normalized values (0–1) for visual comparison; numerical labels above each bar indicate the original index values. Shannon–Wiener (H′) = 2.55; Simpson (1-D) = 0.89; Pielou (J′) = 0.72; Margalef (DMg) = 5.47. Color codes: green = high level; orange = medium level.

Species diversity analysis of Component II revealed a community of 35 species and 498 individuals. The Shannon–Wiener index (H′ = 2.55) indicated intermediate diversity characteristic of forests in intermediate succession; the Margalef index (DMg = 5.47) evidenced high species richness relative to sample size. Community structure exhibited low dominance (Simpson 1-D = 0.89; Pielou J′ = 0.72; Berger–Parker d = 0.17), confirming a relatively homogeneous distribution of abundances among the 35 sampled species (Figure 2).

#### Structural Sampling Completeness: Rarefaction and Asymptotic Estimation

**Figure 3:**
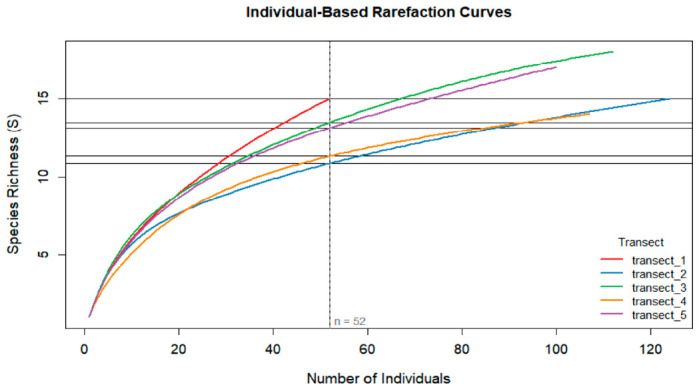
Individual-based rarefaction curves for the five 50 × 4 m transects sampled in the El Mangón remnant, standardized to *n* = 52 individuals (smallest transect size; vertical dashed line). Each curve represents the expected species richness as a function of the number of sampled individuals.

Individual-based rarefaction analysis revealed differences in standardized richness among the five transects. Standardizing to *n* = 52 individuals (smallest transect size: transect 1), rarefied richness ranged from 10.85 spp. (transect 2; SE = 1.35) to 15.00 spp. (transect 1; reference), with intermediate values for transect 3 (13.46 ± 1.51), transect 5 (13.11 ± 1.40), and transect 4 (11.31 ± 1.19) (Figure 3).

**Figure 4:**
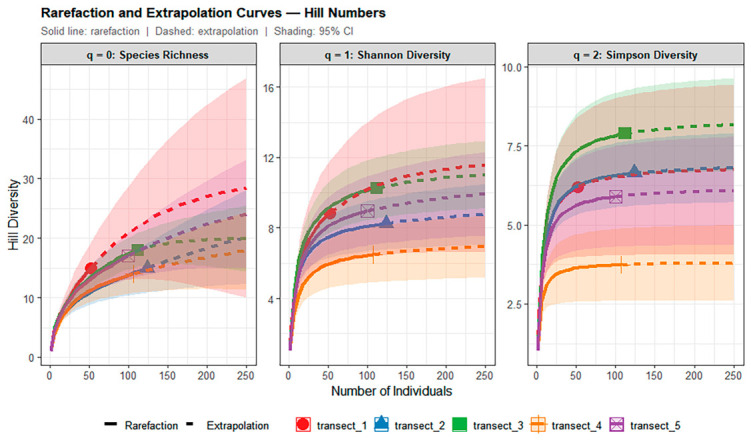
Rarefaction (solid lines) and extrapolation (dashed lines) curves of Hill numbers (q = 0, q = 1, q = 2) for the five 50 × 4 m transects of the El Mangón remnant. q = 0: species richness; q = 1: exponential of Shannon entropy; q = 2: inverse Simpson concentration. Shaded bands represent 95% bootstrap confidence intervals (999 iterations). iNEXT package [21] in R v4.3.0 [22].

Asymptotic extrapolation curves (Hill numbers, q = 0) showed that none of the five transects reached inventory saturation at the individual sampling unit scale: estimated asymptotic richness exceeded observed richness in all cases. Asymptotic estimates ranged from 20.1 spp. (transect 3; observed: 18; 95% CI: 18.0–34.1) to 32.9 spp. (transect 2; observed: 15; 95% CI: 15.0–58.7). Transect 3 was closest to saturation (relative completeness ≈ 90%), while transect 1 and transect 2 showed the largest gaps (completeness ≈ 49% and ≈ 46%, respectively; Table 3, Figure 4). These results confirm that sampling by individual transect is incomplete; the five transects combined allow adequate estimation of community patterns for the objectives of the present study. For the pooled structural sample (498 individuals, 35 species; 12 singletons, 4 doubletons), sample coverage was Ĉ = 97.6% and asymptotic richness (Chao1, q = 0) was 48.2 species, indicating that the combined transects captured the majority of the species expected at this DBH threshold even though individual transects did not saturate.

### 3.3. Horizontal Structure of the Forest Remnant (Component II)

**Figure 5:**
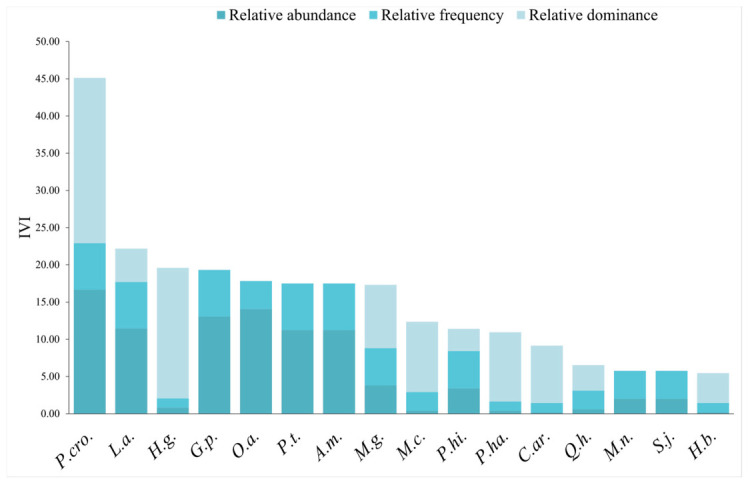
Importance Value Index (IVI) of the sixteen most ecologically dominant species in the El Mangón forest remnant (Component II; *n* = 498 individuals; 35 species). *P.cro.* = *Palicourea crocea* · *L.a.* = *Lacistema aggregatum* · *H.g.* = *Heliconia griggsiana* · *G.p.* = *Geonoma pinnatifrons* · *O.a.* = *Olmedia aspera* · *P.t.* = *Palicourea thyrsiflora* · *A.m.* = *Aiouea montana* · *M.g.* = *Myrsine guianensis* · *M.c.* = *Myrsine coriacea* · *P.hi.* = *Piper hispidum* · *P.ha.* = *Piper hartwegianum* · *C.ar.* = *Coffea arabica* · *Q.h.* = *Quercus humboldtii* · *M.n.* = *Miconia notabilis* · *S.j.* = *Syzygium jambos* · *H.b.* = *Hedyosmum bonplandianum*.

**Figure 6:**
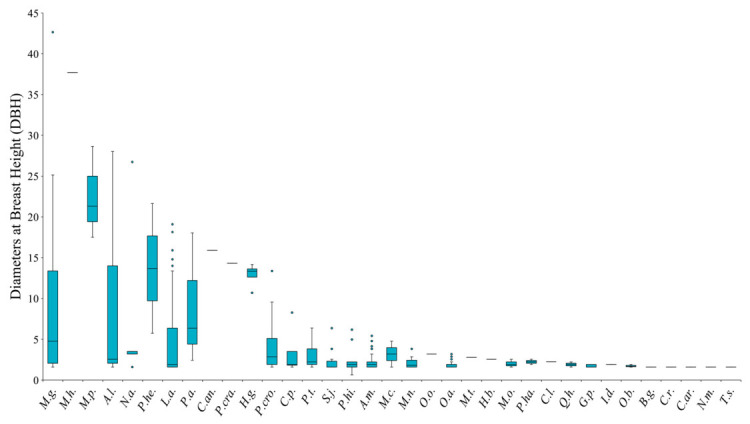
Box plots of diameter at breast height (DBH, cm) for the 35 species recorded in the structural sampling (Component II; *n* = 498 individuals). Boxes show the interquartile range; horizontal lines denote the median; whiskers extend to 1.5 × IQR; points indicate outliers. *Myrcianthes hallii*, *Myrcia popayanensis*, and *Palicourea heterochroma* show the largest median DBH; small-stemmed species cluster on the right of the panel. *A.l.* = *Alchornea latifolia · A.m.* = *Aiouea montana · B.g.* = *Banara guianensis · C.an.* = *Cecropia angustifolia · C.ar.* = *Coffea arabica · C.l.* = *Costus laevis · C.p.* = *Cinchona pubescens · C.r.* = *Citrus reticulata · G.p.* = *Geonoma pinnatifrons · H.b.* = *Hedyosmum bonplandianum · H.g.* = *Heliconia griggsiana · I.d.* = *Inga densiflora · L.a.* = *Lacistema aggregatum · M.c.* = *Myrsine coriacea · M.g.* = *Myrsine guianensis · M.h.* = *Myrcianthes hallii · M.n.* = *Miconia notabilis · M.o.* = *Miconia octona · M.p.* = *Myrcia popayanensis · M.t.* = *Miconia theaezans · N.a.* = *Nectandra acutifolia · N.m.* = *Nectandra mollis · O.a.* = *Olmedia aspera · O.b.* = *Oreopanax bogotensis · O.o.* = *Ocotea oblonga · P.a.* = *Palicourea angustifolia · P.cra.* = *Piper crassinervium · P.cro.* = *Palicourea crocea · P.ha.* = *Piper hartwegianum · P.he.* = *Palicourea heterochroma · P.hi.* = *Piper hispidum · P.t.* = *Palicourea thyrsiflora · Q.h.* = *Quercus humboldtii · S.j.* = *Syzygium jambos · T.s.* = *Toxicodendron striatum*.

Analysis of horizontal structure revealed marked heterogeneity in the ecological importance of species, with IVI values ranging from 1.46 to 45.12 (scale 0–300) (Table A2). *P. crocea* (Rubiaceae) was the ecologically dominant species (IVI = 45.12; relative abundance = 16.67%; relative dominance = 22.21%), followed by *Lacistema aggregatum* (IVI = 22.18) and *Heliconia griggsiana* (IVI = 19.60). The fifteen most important species contributed approximately 80% of total IVI, with the top ten amounting to 66.4% (Figure 5; Table A2). Variation in stem size by species is summarized in Figure 6, where *Myrcianthes hallii, Myrcia popayanensis*, and *Palicourea heterochroma* displayed the largest median DBH values, while shrubby and palm species (*Geonoma pinnatifrons*, *Coffea arabica*, *Citrus reticulata*) presented the smallest size classes.

Of the species, 57.14% (20 spp.) exhibited *G*a < 1 (low-aggregation distribution), while 25.71% (9 spp.) showed an aggregated pattern (*G*a > 2) (Table A2). *Olmedia aspera* showed the highest degree of aggregation (*G*a = 15.28), followed by *P. crocea* (*G*a = 3.60) and *G. pinnatifrons* (*G*a = 2.82). The mixture coefficient (CM = 0.070; 1/CM = 14.23) classified the ecosystem as structurally homogeneous.

### 3.4. Vertical Structure of the Forest Remnant (Component II)

The vertical structure revealed four clearly differentiated strata: the lower stratum (0–8 m), middle stratum (8.1–12 m), upper stratum (12.1–18 m), and emergent stratum (18.1–33 m). The Pretzsch Index reached A = 3.21, equivalent to 64.93% of the theoretical maximum (4.94), indicating notable vertical structural complexity for a forest in active regeneration.

**Figure 7:**
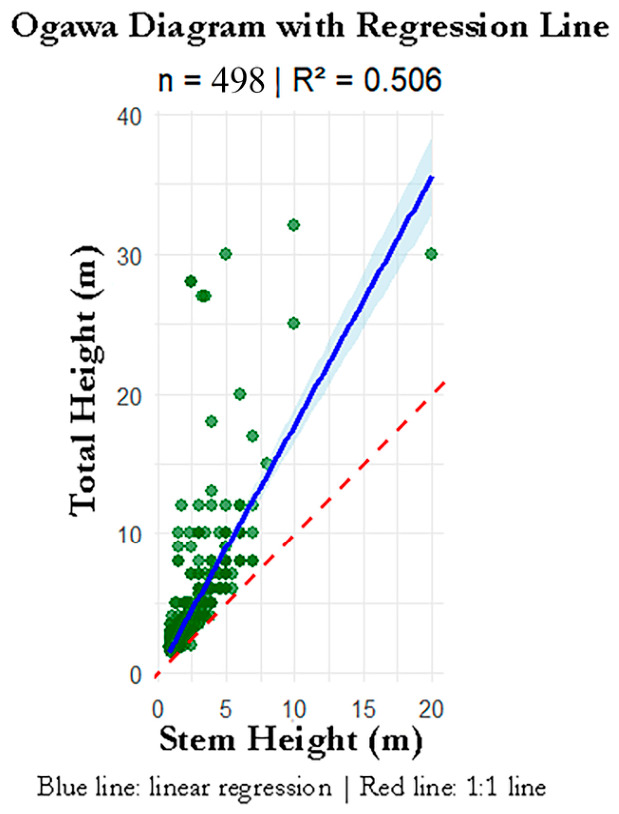
Ogawa diagram with linear regression for the El Mangón forest remnant (*n* = 498 individuals; R^2^ = 0.51). Total height (Ht, m) is plotted against stem height (Hf, m). Solid blue line: linear regression; shaded band: 95% confidence interval; dashed red line: 1:1 reference line. The diagram reveals an asymmetric height distribution with strong concentration of individuals in lower and intermediate classes.

**Figure 8:**
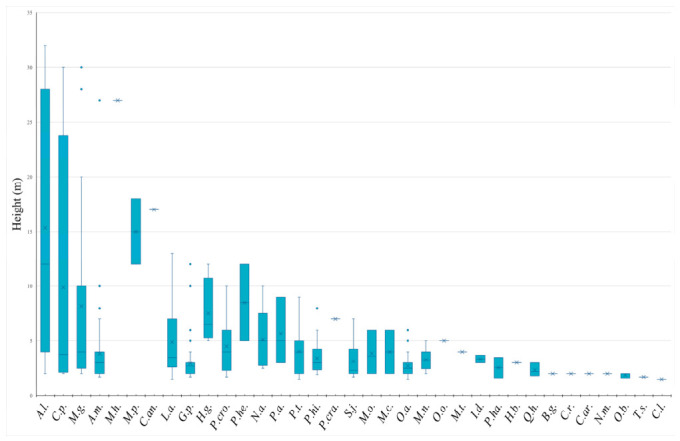
Box plots of total height (Ht, m) by species recorded in the structural sampling of El Mangón (Component II; *n* = 498 individuals). Boxes show the interquartile range; horizontal lines denote the median; whiskers extend to 1.5 × IQR; points indicate outliers. *A.l.* = *Alchornea latifolia · A.m.* = *Aiouea montana · B.g.* = *Banara guianensis · C.an.* = *Cecropia angustifolia · C.ar.* = *Coffea arabica · C.l.* = *Costus laevis · C.p.* = *Cinchona pubescens · C.r.* = *Citrus reticulata · G.p.* = *Geonoma pinnatifrons · H.b.* = *Hedyosmum bonplandianum · H.g.* = *Heliconia griggsiana · I.d.* = *Inga densiflora · L.a.* = *Lacistema aggregatum · M.c.* = *Myrsine coriacea · M.g.* = *Myrsine guianensis · M.h.* = *Myrcianthes hallii · M.n.* = *Miconia notabilis · M.o.* = *Miconia octona · M.p.* = *Myrcia popayanensis · M.t.* = *Miconia theaezans · N.a.* = *Nectandra acutifolia · N.m.* = *Nectandra mollis · O.a.* = *Olmedia aspera · O.b.* = *Oreopanax bogotensis · O.o.* = *Ocotea oblonga · P.a.* = *Palicourea angustifolia · P.cra.* = *Piper crassinervium · P.cro.* = *Palicourea crocea · P.ha.* = *Piper hartwegianum · P.he.* = *Palicourea heterochroma · P.hi.* = *Piper hispidum · P.t.* = *Palicourea thyrsiflora · Q.h.* = *Quercus humboldtii · S.j.* = *Syzygium jambos · T.s.* = *Toxicodendron striatum.*

The Ogawa diagram (Figure 7) showed an asymmetric height distribution, with a strong concentration of individuals in lower and intermediate classes and a small number of dominant canopy trees. Mean height was 4.34 m (±4.09 m). The allometric relationship between Ht and Hf was moderate but significant (R^2^ = 0.51, *p* < 0.001). The lower stratum concentrated 91.77% of individuals (457 ind. of 32 spp.); the middle stratum 5.62% (28 ind. of 12 spp.); and the upper and emergent strata 2.61% (13 ind. of 8 spp.). The species-level distribution of total height (Figure 8) reveals that the tallest medians correspond to canopy trees such as *Alchornea latifolia*, *Cinchona pubescens*, and *M. popayanensis*, while the bulk of the assemblage exhibits modal heights below 5 m, consistent with an inverse-J pattern typical of secondary forests in active regeneration.

On a per-hectare basis, the stand had a basal area of 22.6 m^2^·ha^−1^, a commercial stem volume of 79.23 m^3^·ha^−1^ and a total volume with bark of 264.62 m^3^·ha^−1^ (the previously reported 5.49 and 24.00 m^3^·ha^−1^ were affected by a scaling error in the volume computation); *P. crocea* contributed the greatest number of stems, whereas the largest individual volumes corresponded to a few large canopy trees.

### 3.5. Biomass and Carbon of the Remnant (Component II)

**Figure 9:**
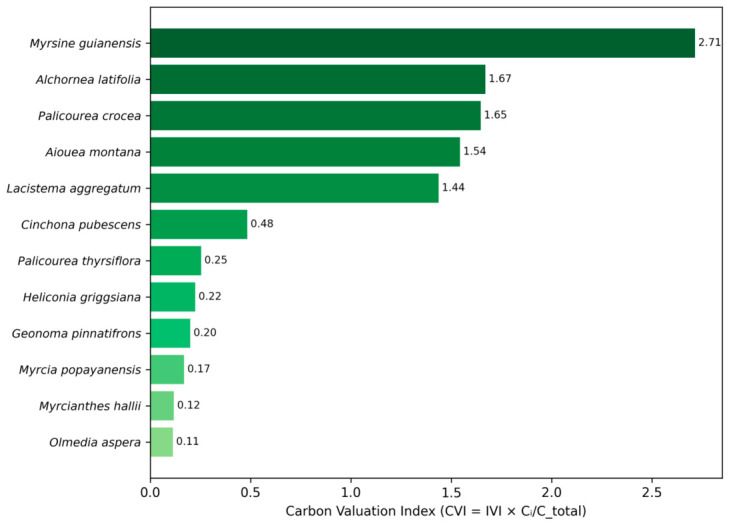
Carbon Valuation Index (CVI) of the most relevant species in the El Mangón remnant (Component II), calculated according to Equation (12). *Myrsine guianensis* leads the index (CVI = 2.71), followed by *Alchornea latifolia* (1.67) and *Palicourea crocea* (1.65). Numerical labels indicate the CVI value of each species.

**Figure 10:**
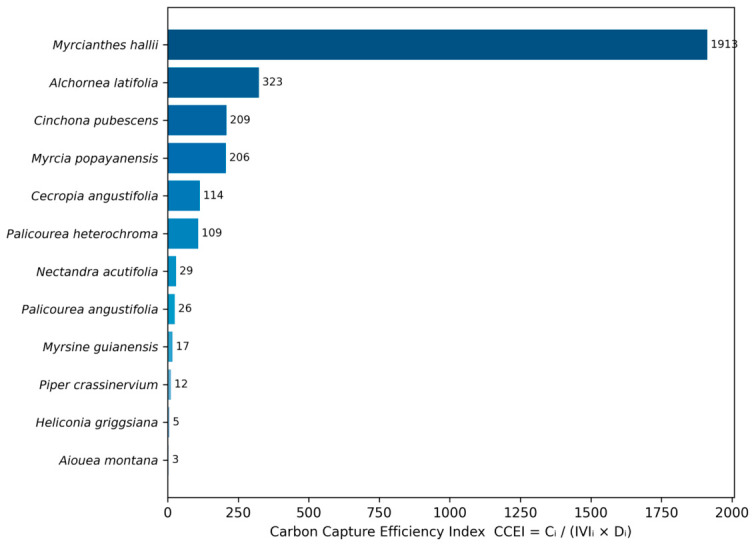
Carbon Capture Efficiency Index (CCEI = Carbon/(IVI × Density)) of the species with the highest values in the El Mangón remnant, calculated according to Equation (13). With aboveground carbon re-estimated using the [28] model, the highest CCEI values correspond to rare, large-stemmed native species (*Myrcianthes hallii*, *Alchornea latifolia*, *Cinchona pubescens*); a high CCEI flags low-density, physiologically efficient storers irrespective of native status (see Section 4.5).

Aboveground carbon, re-estimated with the [28] pantropical allometric model from individual diameter, total height and regional wood density (WD = 0.6 t·m^−3^), totaled 72.85 Mg C·ha^−1^ (72,850 kg·ha^−1^); a wood-density sensitivity analysis (0.4–0.8 t·m^−3^) gave 49.0–96.5 Mg C·ha^−1^. *P. crocea* (Rubiaceae) showed the highest structural dominance (IVI = 45.12), but under the corrected carbon the highest CVI corresponds to *Myrsine guianensis* (CVI = 2.71), followed by *Alchornea latifolia* (1.67) and *P. crocea* (1.65) (Figure 9). By contrast, the Carbon Capture Efficiency Index (CCEI) highlighted species with high carbon storage per unit of ecological importance and density, mainly rare or low-density large-stemmed species (Figure 10). Aboveground carbon is dominated by a few large canopy trees (*A. latifolia*, 32%; *M. guianensis*, 16%; *C. pubescens*, 10.5%), whereas *P. crocea* contributes only 3.6%.

Note on *C. arabica*: this cultivated species, native to Ethiopia and introduced to Colombia during the colonial period [32], occurs in the transects as remnant individuals from a coffee plantation with more than 50 years of history at the site, currently abandoned. *C. arabica* is therefore not a native component of the Colombian sub-Andean ecosystem. Its high CCEI value reflects specific physiological characteristics (photosynthetic efficiency under shade and high C/N ratio) rather than a central ecological function in the natural ecosystem; ecological implications are discussed in Section 4.5.

**Figure 11:**
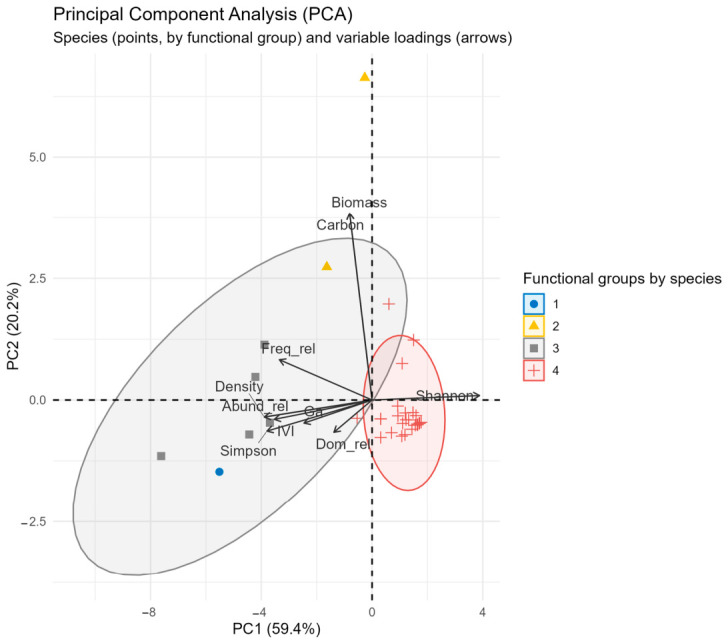
Principal Component Analysis (PCA) biplot of structural and functional variables for the species recorded in Component II. PC1 = 59.4% of variance; PC2 = 20.2% (cumulative = 79.6%), computed on ten standardized variables (biomass, carbon, IVI, relative abundance, relative frequency, relative dominance, Shannon and Simpson contributions, Pielou Ga, density; mean = 0, SD = 1) with carbon re-estimated using [28]. Colored ellipses indicate the four functional groups identified by k-means partitioning.

Functional-group analysis (k-means, four groups) on the corrected carbon separated a large group of low-carbon species (24 spp.; mean ≈ 192 kg·ha^−1^) from small groups of high-carbon, large-stemmed trees (a group dominated by *A. latifolia*, ≈ 23,456 kg·ha^−1^, and a high-storage group with mean ≈ 7178 kg·ha^−1^); *P. crocea* (83 ind.) fell in a high-density, moderate-carbon group. PCA on the ten standardized variables showed that PC1 (59.4% of variance) was influenced primarily by IVI, density, relative abundance and the diversity contributions, while PC2 (20.2%; cumulative 79.6%) was associated with biomass, carbon and relative dominance (Figure 11).

### 3.6. Carbon Sustainability Index (CSI) by Spatial Pattern (Component II)

**Figure 12:**
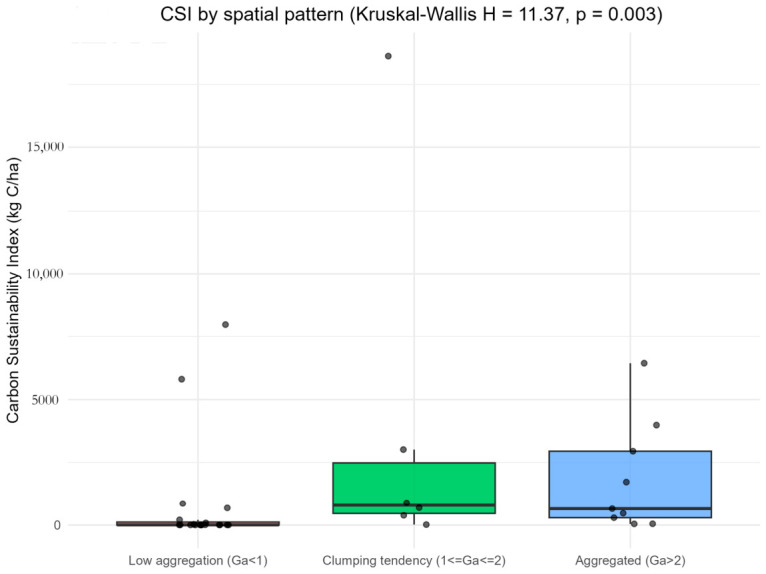
Carbon Sustainability Index (CSI) according to spatial-pattern group in the El Mangón remnant. Boxes show the distribution of individual species CSI values for three groups: aggregated/grouped (Ga > 2; 9 spp.), dispersed/low aggregation (Ga < 1; 20 spp.), and species with tendency toward grouping (1 ≤ Ga ≤ 2). Boxes show interquartile range; horizontal lines indicate the median; whiskers extend to 1.5 × IQR; points are outliers. Differences among groups were statistically significant (Kruskal–Wallis, *p* = 0.003), the low-aggregation group showing lower CSI than the clumping-tendency and aggregated groups (corrected carbon, [28]).

Evaluation of the CSI by spatial pattern revealed differences in cumulative values among distribution groups (Figure 12). With carbon re-estimated using [28], the low-aggregation group (20 spp.) showed the lowest mean CSI (785 kg C·ha^−1^ per species), whereas the clumping-tendency (6 spp.; mean 3942) and aggregated (9 spp.; mean 1846) groups—which include the large canopy trees—showed higher values.

The Kruskal–Wallis test detected statistically significant differences in individual CSI values among the three spatial-pattern groups (H = 11.37, df = 2, *p* = 0.003). Post hoc pairwise comparisons (Wilcoxon with Benjamini–Hochberg correction) showed that the low-aggregation group differed significantly from both the clumping-tendency and the aggregated groups (*p* = 0.017 in both cases), whereas the latter two did not differ (*p* = 0.86). The mean CSI per species was lowest in the low-aggregation group (785 kg C·ha^−1^) and higher in the clumping-tendency (3942) and aggregated (1846) groups, indicating that most sustainable carbon is held by a few large, spatially concentrated trees.

## 4. Discussion

### 4.1. Taxonomic Diversity and Floristic Richness

The floristic richness of the El Mangón remnant (281 spp., 209 gen., 99 fam.) is comparable to that reported for El Peñol, Antioquia (285 spp.; [1]), and surpasses records from other Cauca remnants such as Timbío, Hacienda Hato Viejo (151 spp.; [11]) and Popayán, Reserva Forestal Cajete (164 spp.; [3]). This level of richness confirms the patterns of high diversity characteristic of the sub-Andean forests of the Colombian Central Cordillera described by [7,9,33].

The predominance of Poaceae (20 spp.), Asteraceae (13 spp.), and Orchidaceae (13 spp.) partially departs from the general pattern of Colombian sub-Andean forests, where Rubiaceae and Melastomataceae typically dominate floristic composition [7,9]. This peculiarity may be explained by the specific ecological conditions of the site—especially the surrounding agricultural matrix and the disturbance history—which favor the establishment of grasses and composites typical of forest edges and intermediate successional stages [8]. The dominance of Rubiaceae in the structural analyses (driven mainly by *P. crocea*) indicates that the family is indeed important at the structural level, although not in terms of species diversity in the general (non-systematic) inventory.

The high representation of epiphytes (44.13%) greatly exceeds the typical values of 25–35% reported for Neotropical sub-Andean forests [34]. This exceptional epiphytic richness can be attributed to: the permanence of forest cover since 1912 without clear-cutting, the presence of four well-differentiated vertical strata that increase microhabitat availability, and microclimate stability provided by the three water bodies crossing the remnant. The association between canopy vertical structural heterogeneity and epiphytic diversity has been documented in tropical Andean montane forests [10,35]. Because the general (non-systematic) inventory deliberately emphasized epiphytes, lianas and ferns, this proportion partly reflects targeted collecting effort and is not directly comparable with epiphyte proportions derived from systematic plot-based sampling.

### 4.2. Diversity Indices and Community Structure

The Shannon–Wiener index (H′ = 2.55) places El Mangón in an intermediate range relative to other Colombian sub-Andean forests, below values reported for Puracé (H′ = 3.0; [12]) and Santander de Quilichao (H′ = 3.0; [13]), but above records from Timbío (H′ = 2.0; [14]) and Nariño (H′ = 2.0; [6]). Pielou’s evenness (J′ = 0.72) indicates a moderately uniform abundance distribution, consistent with an ecosystem in successional reorganization.

Patterns of intermediate diversity and low dominance (Simpson 1-D = 0.89; Berger–Parker d = 0.17) are consistent with post-disturbance recovery dynamics described by [15] for tropical Andean forests in intermediate succession. The high Margalef index (DMg = 5.47) reflects high species richness relative to the structural sample size (0.1 ha).

### 4.3. Horizontal Structure and Spatial-Distribution Patterns

The marked dominance of *P. crocea* (IVI = 45.12) constitutes an atypical pattern for sub-Andean forests. The concentration of 66.4% of total IVI in ten species coincides with patterns recorded in fragmented sub-Andean remnants, where the first twelve species typically accumulate ≈72% of IVI [16,17]. This pattern may be partly attributable to the placement of transects in areas of higher density of *P. crocea* (Ga = 3.60, aggregated pattern), an aspect that constitutes a methodological limitation discussed in Section 4.6.

The predominance of low-aggregation patterns (57.14% of species with Ga < 1) contrasts with the predominantly aggregated pattern of mature tropical forests and suggests active interspecific competition and seed-dispersal limitations, processes typical of intermediate successional stages [15,18]. The high aggregation of *O. aspera* (Ga = 15.28) and *P. crocea* (Ga = 3.60) indicates specific reproductive strategies and marked microenvironmental preferences.

### 4.4. Vertical Structure and Successional Position

The presence of four well-differentiated vertical strata and a Pretzsch Index of 3.21 (64.93% of the theoretical maximum) indicates notable vertical structural complexity for a forest in active regeneration. This value is consistent with the three to four well-defined strata reported for mature secondary forests of the Colombian Andes in advanced passive restoration [3,36], where gradual canopy differentiation generates light gradients that diversify microhabitats for recovering species.

The concentration of 91.77% of individuals in the lower stratum (0–8 m) reflects typical dynamics of recovering forests, where active regeneration generates high densities of young individuals [15,37]. The inverse-J pattern in diameter and height distributions is characteristic of communities with continuous recruitment and density-dependent mortality, suggesting a successional trajectory toward a forest with greater canopy dominance. The Ht–Hf allometric relationship (R^2^ = 0.51, *p* < 0.001) indicates the architectural heterogeneity expected in secondary forests with high diversity of growth forms [37].

### 4.5. Carbon Storage and Sustainability

Carbon-storage patterns revealed marked functional heterogeneity among species. When aboveground biomass is re-estimated with the [28] pantropical model, total aboveground carbon is 72.85 Mg C·ha^−1^—within the range reported for Andean montane secondary forests (≈20–80 Mg C·ha^−1^) and broadly consistent with secondary forests 25–30 years old accumulating 40–70% of the carbon of comparable mature stands [17,18]. Carbon is concentrated in a few large canopy trees (*A. latifolia*, *M. guianensis*, *C. pubescens*) rather than in the structurally dominant *P. crocea* (only 3.6% of total), whereas species with lower structural dominance show higher per-individual capture efficiency (CCEI). The much lower value initially obtained with the volumetric route (2.87 Mg C·ha^−1^) reflected an under-estimation of standing volume and is not retained. This result also reinforces the importance of selecting appropriate allometric models for biomass and carbon estimation, since species-specific or locally calibrated equations can substantially improve the accuracy of aboveground biomass estimates [38].

The presence of *C. arabica* (L.) among the species with a relatively high CCEI requires careful interpretation. The presence of this species in the transects corresponds to remnant individuals from a coffee plantation with more than 50 years of history at the site, currently abandoned. *C. arabica*, native to the Ethiopian highlands and introduced to Colombia during the colonial period [32], is not a native component of the Colombian sub-Andean ecosystem. Its maintenance under the canopy of the remnant—without reproducing or expanding as part of the native assemblage—and its high CCEI may reflect specific physiological characteristics (photosynthetic efficiency under shade conditions; high C/N ratio) rather than a central ecological function in ecosystem carbon storage. This distinction is relevant both for the interpretation of the carbon analysis and for site management within the framework of the RNSC declaration, where the status of introduced species must be formally evaluated. More generally, this case delimits the interpretation of CCEI: because the index rewards high carbon per unit of ecological importance and density, it can be maximized by low-density, physiologically efficient species—including introduced ones—irrespective of their conservation value, so a high CCEI must not be read as ecological priority. Accordingly, within the prospective RNSC management plan, *C. arabica* should be treated as a non-native legacy of former cultivation: enrichment planting and restoration should rely on native species, whereas isolated, non-regenerating coffee individuals under the canopy may be tolerated and monitored rather than actively propagated, with gradual removal prioritized only where regeneration or expansion is detected.

Functional-group analyses and PCA revealed clear trade-offs between diversity and carbon storage: the group dominated by *P. crocea* maximizes total storage but reduces structural diversity, while groups with a greater number of species generate greater functional heterogeneity. With the corrected carbon, the CSI differs significantly among spatial-distribution groups (Kruskal–Wallis, *p* = 0.003): aggregated and clumping-tendency species—which include the large canopy trees that store most carbon—show a higher CSI than low-aggregation species. In this stand, therefore, carbon sustainability is associated with the spatial concentration of a few large trees rather than with low-aggregation patterns, broadly consistent with evidence that the spatial arrangement of tree species influences forest functioning [39]—although that study found spatial mixing, rather than aggregation, to enhance functioning, so this single-site association more likely reflects the large carbon mass held by a few aggregated trees; this single-site, single-time result nonetheless requires validation with greater sampling intensity and temporal monitoring. Beyond these descriptive results, the three indices are intended to provide information that IVI, biomass, carbon and ordination do not yield individually: CVI identifies species whose conservation simultaneously protects a large, structurally anchored carbon pool; CCEI is an intensive (per-importance, per capita) measure that flags efficient storers for potential enrichment planting, subject to the native-status filter noted above; and CSI weights each species’ carbon by its spatial pattern and diversity contribution as a first-order proxy for how spatially buffered that carbon is. Their shared assumptions are made explicit: carbon rests on a single regional wood-density value (Section 2.7), so species-level scores are provisional; the indices are single-site, single-time descriptors rather than validated predictors; and the CSI spatial factor is an ordinal weighting, not a mechanistic stability model. They therefore complement—and do not replace—IVI, biomass and ordination, and require multi-site validation before generalization. It should also be noted that, because absolute stored carbon varies among species by orders of magnitude, it dominates the CSI despite the lower spatial weight assigned to aggregated species; the index therefore mainly indicates where carbon is concentrated rather than how evenly it is spatially buffered, which should be borne in mind when interpreting the present results.

### 4.6. Study Limitations

This study presents limitations that must be considered in the interpretation of results. First, the structural sampling intensity (0.1 ha out of 24 ha, equivalent to 0.42%) is low for a forest with the reported spatial heterogeneity. The high abundance of *P. crocea* (IVI = 45.12) may partly reflect a bias of the transects toward areas of higher density of this species. Future studies with greater sampling intensity or stratified sampling designs will allow evaluation of the representativeness of the structural analysis.

Second, biomass estimation with a uniform WD value (0.6 t·m^−3^) may introduce biases in the comparative estimation of carbon by species; although aboveground biomass was estimated with [28] pantropical model, the use of species-specific wood densities (e.g., from a global wood-density database) rather than a single regional value would further reduce this uncertainty. Third, the rarefaction analysis indicated that sampling by individual transect was incomplete (relative completeness between ≈46% and ≈90%), reinforcing the need to expand the structural inventory. Finally, the CVI, CCEI, and CSI indices proposed here are novel tools that require comparative validation at additional sites before generalizing their applicability as indicators of forest carbon sustainability.

## 5. Conclusions

The El Mangón forest remnant represents one of the floristically richest sub-Andean fragments documented in the department of Cauca and in southwestern Colombia, with a species diversity that reflects the conservation potential of secondary forests with a prolonged history of private protection. The composition of the general inventory—with predominance of epiphytes (44.13%) and significant cryptogamic contributions, including bryophytes and lichens—is consistent with a long-protected, structurally complex canopy. In this intensive agricultural landscape, descriptors such as “microclimate maturity” and “biodiversity refuge” are advanced as hypotheses to be tested with expanded structural sampling, rather than as established status.

Structural analysis indicates an actively regenerating, low-biomass interior stand—four differentiated vertical strata, a Pretzsch Index of 3.21 (64.93% of the theoretical maximum), and an inverse-J size structure with most stems below 8 m and low basal area—consistent with an early-to-intermediate successional phase rather than a mature forest. The structural dominance of *P. crocea* and the predominance of low-aggregation spatial-distribution patterns are indicators of a community in active competitive reorganization, compatible with a trajectory toward stages of greater forest complexity.

The proposed carbon indices (CVI, CCEI, and CSI) constitute complementary tools to standard floristic and structural analysis by integrating the magnitude of carbon storage with individual species efficiency and the stability associated with their spatial-distribution patterns. Aboveground carbon (72.85 Mg C·ha^−1^, estimated with [28]) is concentrated in a few large canopy trees, and the CSI differs significantly among spatial-distribution groups (*p* = 0.003), being higher in aggregated, large-stemmed species; this single-site result requires validation with greater sampling intensity and temporal monitoring.

The potential declaration of the property as a Natural Reserve of Civil Society has a solid ecological basis: the documented floristic richness, the complex vertical structure, the carbon-storage potential, and the uninterrupted history of private conservation justify inclusion of the remnant in national biodiversity-protection instruments. Technical and legal support for the Corporación Autónoma Regional del Cauca is recommended for the declaration process as a landscape-conservation strategy in the municipality of Piendamó.

This contribution helps fill a floristic gap in the Colombian Central Cordillera and provides a quantitative baseline for long-term monitoring. For future research, we recommend: (i) increasing the intensity of structural sampling through stratified designs; (ii) updating the biomass estimation model with species-specific wood densities; (iii) formally documenting the status of introduced species present in the remnant; and (iv) evaluating the connectivity potential of the remnant with other forest fragments in the Río Piendamó corridor.

## Figures and Tables

**Table 1:** Spatial factor (*F*spatial) applied in the calculation of the Carbon Sustainability Index (CSI) according to the Pielou aggregation index (*G*a).

Distribution Pattern (*G*a)	*F*spatial	Criterion
Low aggregation (*G*a < 1)	1.00	Dispersed/regular distribution
Tendency toward clumping (1 ≤ *G*a ≤ 2)	0.75	Intermediate pattern
Aggregated (*G*a > 2)	0.50	Aggregated pattern
Other cases	0.75	Default value

**Table 2:** Ten most diverse plant families recorded in the El Mangón sub-Andean forest remnant (Component I, general (non-systematic) floristic inventory across 24 ha). Total richness = 281 species in 99 families.

Family	Species	% of Total
Poaceae	20	7.12
Asteraceae	13	4.63
Orchidaceae	13	4.63
Polypodiaceae	10	3.56
Rubiaceae	10	3.56
Piperaceae	9	3.20
Fabaceae	8	2.85
Melastomataceae	8	2.85
Bromeliaceae	7	2.49
Bryaceae	7	2.49
Subtotal (10 most diverse families)	105	37.40
Total	281	100.00

**Table 3:** Observed, rarefied (*n* = 52), and asymptotically estimated richness (Hill numbers, q = 0) by transect. Relative completeness = observed S/estimated asymptotic S. Analysis with iNEXT [21] in R v4.3.0 [22].

Transect	N	S obs.	S raref. (*n* = 52)	SE	S asympt.	95% CI	Complet.
Transect 1	52	15	15.00	0.000	30.7	15.0–64.4	≈49%
Transect 2	124	15	10.85	1.348	32.9	15.0–58.7	≈46%
Transect 3	112	18	13.46	1.511	20.1	18.0–34.1	≈90%
Transect 4	107	14	11.31	1.185	21.9	14.0–39.0	≈64%
Transect 5	100	17	13.11	1.398	29.1	17.0–53.0	≈58%

## Data Availability

The data supporting the findings of this study are available from the corresponding author (L.E.L.-V.) upon reasonable request. The complete floristic inventory is provided as (Appendix A).

## Abbreviations

The following abbreviations are used in this manuscript:

AGB	Aboveground biomass
ANLA	Autoridad Nacional de Licencias Ambientales
BA	Basal area
BEF	Biomass expansion factor
CAUP	Herbario de la Universidad del Cauca
CBH	Circumference at breast height
CCEI	Carbon Capture Efficiency Index
CSI	Carbon Sustainability Index
CVI	Carbon Valuation Index
DBH	Diameter at breast height; D

## Appendix A

Complete floristic inventory of the El Mangón sub-Andean forest remnant (Tunía, Piendamó, Cauca, Colombia), comprising 281 species in 209 genera and 99 families, with taxonomic classification and growth-form designation.

**Table A1:** Complete floristic inventory of the El Mangón sub-Andean forest remnant (Component I; general (non-systematic) collections across 24 ha; 281 species in 209 genera and 99 families). Species are listed alphabetically by family.

Family	Species
Acanthaceae	*Dianthera secunda*, *Lepidagathis alopecuroidea*, *Ruellia blechum*
Actinidiaceae	*Saurauia scabra*
Amaranthaceae	*Alternanthera porrigens*, *Alternanthera sessilis*, *Iresine diffusa*
Anacardiaceae	*Mauria heterophylla*, *Toxicodendron striatum*
Araceae	*Anthurium longistamineum*, *Anthurium microspadix*
Araliaceae	*Oreopanax bogotensis*
Arecaceae	*Geonoma pinnatifrons*
Arthoniaceae	*Arthonia* sp. 1, *Herpothallon rubrocinctum*, *Herpothallon rubroechinatum*
Aspleniaceae	*Asplenium aethiopicum*, *Asplenium theciferum*
Asteraceae	*Acmella ciliata*, *Ageratum conyzoides*, *Austroeupatorium inulifolium*, *Baccharis latifolia*, *Baccharis trinervis*, *Bidens pilosa*, *Calea colombiana*, *Calea sessiliflora*, *Chromolaena laevigata*, *Critoniella acuminata*, *Elephantopus mollis*, *Pseudelephantopus spiralis*, *Sonchus asper*
Bacidiaceae	*Bacidia biatorina*
Bartramiaceae	*Breutelia chrysea*
Brachytheciaceae	*Brachythecium plumosum*, *Brachythecium stereopoma*
Brassicaceae	*Lepidium trianae*
Bromeliaceae	*Cipuropsis capituligera*, *Racinaea fraseri*, *Racinaea penlandii*, *Racinaea tenuispica*, *Tillandsia complanata*, *Tillandsia fendleri*, *Tillandsia myriantha*
Bryaceae	*Anomobryum conicum*, *Anomobryum julaceum*, *Brachymenium globosum*, *Bryum andicola*, *Bryum argenteum*, *Bryum densifolium*, *Rhodobryum beyrichianum*
Cactaceae	*Rhipsalis sulcata*
Caliciaceae	*Dirinaria applanata*, *Dirinaria confluens*, *Pyxine cocoes*
Callicostaceae	*Hypnella diversifolia*
Calymperaceae	*Syrrhopodon gaudichaudii*
Campanulaceae	*Centropogon lehmannii*
Candelariaceae	*Candelaria concolor*
Chloranthaceae	*Hedyosmum bonplandianum*, *Hedyosmum goudotianum*
Coccocarpiaceae	*Coccocarpia* sp.
Coenogoniaceae	*Coenogonium linkii*, *Coenogonium pinetti*
Collemataceae	*Leptogium granulatum*, *Leptogium phyllocarpum*
Cordiaceae	*Varronia acuta*
Costaceae	*Costus laevis*
Cryphaeaceae	*Cryphaea patens*, *Cryphaea ramosa*
Cucurbitaceae	*Melothria pendula*
Cyatheaceae	*Cyathea horrida*
Cyperaceae	*Cyperus hermaphroditus*, *Rhynchospora hieronymi*, *Rhynchospora nervosa*
Daltoniaceae	*Adelothecium bogotense*
Dicranaceae	*Bryohumbertia filifolia*, *Campylopus pilifer*, *Dicranella hilariana*, *Holomitrium flexuosum*, *Leucoloma cruegerianum*
Dryopteridaceae	*Elaphoglossum burchellii*, *Elaphoglossum ellipsoideum*, *Elaphoglossum muscosum*, *Elaphoglossum paleaceum*, *Peltapteris flabellata*, *Polystichum platyphyllum*
Euphorbiaceae	*Alchornea latifolia*, *Croton hibiscifolius*, *Euphorbia hirta*
Fabaceae	*Acaciella angustissima*, *Desmodium purpusii*, *Inga densiflora*, *Inga edulis*, *Senna hirsuta*, *Senna pendula*, *Trifolium repens*, *Zornia reticulata*
Fagaceae	*Quercus humboldtii*
Fissidentaceae	*Fissidens asplenioides*, *Fissidens lagenarius*, *Fissidens polypodioides*
Frullaniaceae	*Frullania* sp.
Funariaceae	*Funaria hygrometrica*
Gesneriaceae	*Besleria solanoides*, *Kohleria spicata*
Graphidaceae	*Graphis chondroplaca*
Heliconiaceae	*Heliconia burleana*, *Heliconia gaiboriana*, *Heliconia griggsiana*
Herbertaceae	*Herbertus pensilis*
Hypericaceae	*Vismia lauriformis*
Hypnaceae	*Ctenidium malacodes*, *Ectropothecium leptochaeton*, *Isopterygium tenerifolium*, *Isopterygium tenerum*, *Mittenothamnium reptans*
Hypoxidaceae	*Hypoxis decumbens*
Lacistemataceae	*Lacistema aggregatum*
Lamiaceae	*Clinopodium brownei*, *Mesosphaerum pectinatum*, *Mesosphaerum sidifolium*, *Ocimum campechianum*, *Salvia scutellarioides*, *Salvia tiliifolia*, *Scutellaria incarnata*
Lauraceae	*Aiouea montana*, *Nectandra acutifolia*, *Nectandra mollis*, *Ocotea cuatrecasasii*, *Ocotea oblonga*
Lecanoraceae	*Lecanora argentata*
Lejeuneaceae	*Bryopteris filicina*, *Lejeunea* sp.
Leucobryaceae	*Atractylocarpus longisetus*, *Leucobryum antillarum*, *Leucobryum giganteum*
Loranthaceae	*Oryctanthus spicatus*, *Passovia pyrifolia*
Lycopodiaceae	*Huperzia linifolia*, *Lycopodiella cernua*, *Lycopodium clavatum*
Lythraceae	*Cuphea strigulosa*
Malpighiaceae	*Stigmaphyllon bogotense*
Malvaceae	*Heliocarpus americanus*, *Pavonia sepioides*, *Sida rhombifolia*, *Triumfetta bogotensis*, *Triumfetta rhomboidea*
Marchantiaceae	*Marchantia chenopoda*
Melastomataceae	*Arthrostemma ciliatum*, *Chaetogastra gracilis*, *Miconia desmantha*, *Miconia notabilis*, *Miconia octona*, *Miconia theaezans*, *Miconia versicolor*, *Rhynchanthera mexicana*
Meteoriaceae	*Meteoridium remotifolium*, *Meteorium nigrescens*, *Squamidium leucotrichum*
Mniaceae	*Plagiomnium rhynchophorum*
Moraceae	*Olmedia aspera*
Myrtaceae	*Myrcia popayanensis*, *Myrcianthes hallii*, *Psidium guineense*, *Syzygium jambos*
Octoblepharaceae	*Octoblepharum albidum*
Orchidaceae	*Comparettia falcata*, *Dichaea humilis*, *Dichaea pendula*, *Epidendrum melinanthum*, *Erycina pusilla*, *Lepanthes tracheia*, *Oncidium adelaidae*, *Pleurothallis cordata*, *Prosthechea grammatoglossa*, *Rodriguezia granadensis*, *Stelis argentata*, *Stelis pusilla*, *Trizeuxis falcata*
Orobanchaceae	*Castilleja angustata*
Orthotrichaceae	*Groutiella chimborazensis*, *Groutiella tomentosa*, *Macromitrium guatemaliense*, *Macromitrium* cf. *punctatum*, *Macromitrium richardii*
Pallaviciniaceae	*Symphyogyna brasiliensis*
Pannariaceae	*Parmeliella triptophylla*
Parmeliaceae	*Crespoa crozalsiana*, *Hypotrachyna* sp., *Rimelia subisidiosa*, *Usnea* sp.
Peltigeraceae	*Crocodia aurata*, *Sticta hypoglabra*
Physciaceae	*Leucodermia leucomelos*
Piperaceae	*Peperomia haematolepis*, *Peperomia silvivaga*, *Peperomia tetraphylla*, *Piper auritum*, *Piper capillipes*, *Piper catripense*, *Piper crassinervium*, *Piper hartwegianum*, *Piper hispidum*
Plantaginaceae	*Plantago major*
Poaceae	*Agrostis perennans*, *Andropogon aequatoriensis*, *Chloris radiata*, *Cynodon nlemfuensis*, *Digitaria ciliaris*, *Digitaria horizontalis*, *Eleusine indica*, *Eragrostis bahiensis*, *Homolepis glutinosa*, *Lasiacis divaricata*, *Lasiacis ligulata*, *Lasiacis nigra*, *Lasiacis sorghoidea*, *Oplismenus hirtellus*, *Panicum polygonatum*, *Paspalum candidum*, *Paspalum conjugatum*, *Pseudechinolaena polystachya*, *Steinchisma laxa*, *Zeugites americanus*
Polygalaceae	*Polygala asperuloides*
Polypodiaceae	*Campyloneurum brevifolium*, *Campyloneurum phyllitidis*, *Grammitis apiculata*, *Pecluma plumula*, *Pleopeltis astrolepis*, *Pleopeltis macrocarpa*, *Serpocaulon adnatum*, *Serpocaulon funckii*, *Serpocaulon lasiopus*, *Serpocaulon levigatum*
Primulaceae	*Myrsine coriacea*, *Myrsine guianensis*
Pteridaceae	*Adiantum andicola*, *Polytaenium lineatum*, *Radiovittaria gardneriana*, *Vittaria graminifolia*
Pterobryaceae	*Calyptothecium duplicatum*, *Hildebrandtiella guyanensis*
Ramalinaceae	*Lopezaria versicolor*, *Ramalina celastri*
Roccellaceae	*Bactrospora* sp.
Rosaceae	*Rubus idaeus*, *Rubus urticifolius*
Rubiaceae	*Chiococca alba*, *Cinchona pubescens*, *Coccocypselum lanceolatum*, *Coffea arabica*, *Ladenbergia oblongifolia*, *Palicourea angustifolia*, *Palicourea crocea*, *Palicourea heterochroma*, *Palicourea thyrsiflora*, *Spermacoce capitata*
Rutaceae	*Citrus reticulata*
Salicaceae	*Banara guianensis*
Schizaeaceae	*Anemia hirsuta*, *Anemia villosa*
Selaginellaceae	*Selaginella diffusa*
Sematophyllaceae	*Sematophyllum subpinnatum*
Solanaceae	*Solanum americanum*, *Solanum caripense*, *Solanum quitoense*, *Solanum sisymbriifolium*, *Solanum umbellatum*
Sphagnaceae	*Sphagnum meridense*
Teloschistaceae	*Gallowayella weberi*, *Teloschistes flavicans*
Thuidiaceae	*Thuidium peruvianum*, *Thuidium tomentosum*
Urticaceae	*Cecropia angustifolia*, *Phenax sonneratii*
Verbenaceae	*Verbena litoralis*
Viburnaceae	*Viburnum lehmannii*
Zingiberaceae	*Hedychium coronarium*, *Renealmia ligulata*

**Table A2:** Importance Value Index (IVI), Pielou aggregation index (*G*a), and spatial-distribution interpretation for the 35 species recorded in the structural sampling (Component II, *n* = 498 individuals).

Species	IVI	*G*a	Interpretation
*Aiouea montana*	17.50	2.43	Aggregated
*Alchornea latifolia*	5.18	1.53	Tendency toward clumping
*Banara guianensis*	1.86	0.04	Low aggregation
*Cecropia angustifolia*	2.96	0.90	Low aggregation
*Cinchona pubescens*	4.59	0.87	Low aggregation
*Citrus reticulata*	1.48	0.90	Low aggregation
*Coffea arabica*	9.15	0.90	Low aggregation
*Costus laevis*	1.48	0.90	Low aggregation
*Geonoma pinnatifrons*	19.35	2.82	Aggregated
*Hedyosmum bonplandianum*	5.46	0.90	Low aggregation
*Heliconia griggsiana*	19.60	0.17	Low aggregation
*Inga densiflora*	4.39	0.09	Low aggregation
*Lacistema aggregatum*	22.18	2.48	Aggregated
*Miconia notabilis*	5.77	2.18	Aggregated
*Miconia octona*	3.16	1.17	Tendency toward clumping
*Miconia theaezans*	1.71	0.90	Low aggregation
*Myrcia popayanensis*	3.15	1.17	Tendency toward clumping
*Myrcianthes hallii*	1.50	0.90	Low aggregation
*Myrsine coriacea*	12.36	0.78	Low aggregation
*Myrsine guianensis*	17.31	2.36	Aggregated
*Nectandra acutifolia*	3.92	1.96	Tendency toward clumping
*Nectandra mollis*	2.45	0.90	Low aggregation
*Ocotea oblonga*	2.68	0.90	Low aggregation
*Olmedia aspera*	17.85	15.28	Aggregated
*Oreopanax bogotensis*	5.20	0.65	Low aggregation
*Palicourea angustifolia*	3.25	1.17	Tendency toward clumping
*Palicourea crocea*	45.12	3.60	Aggregated
*Palicourea heterochroma*	2.09	1.79	Tendency toward clumping
*Palicourea thyrsiflora*	17.51	2.43	Aggregated
*Piper crassinervium*	3.67	0.90	Low aggregation
*Piper hartwegianum*	10.95	0.09	Low aggregation
*Piper hispidum*	11.41	0.74	Low aggregation
*Quercus humboldtii*	6.52	0.13	Low aggregation
*Syzygium jambos*	5.76	2.18	Aggregated
*Toxicodendron striatum*	1.46	0.90	Low aggregation
